# Koch’s Lymph and Swan’s Tuberculinum

**Published:** 1891-06

**Authors:** 


### KOCH'S LYMPH AND SWAN'S TUBERCULINUM.

Under the above quoted heading, an editorial appeared in the last number of THE HOMŒOPATHIC PHYSICIAN which should not be allowed to pass into oblivion without a notice. The writer, after commenting upon the experiments of Dr. Koch, telling us of their unscientific character, of their failure to cure, and of their danger of killing, thus continues: "Contrast this with Hahnemann's directions in respect of finding the curative powers of a remedy! On the one hand, we have a hazardous, death-causing mode, on the other a law of nature by which the true physician is able to conquer disease without jeopardizing the health of a patient. Induction, that process of drawing a general conclusion from particular cases, will, in a measure, render the Hahnemannian competent to approximate the curative value of such substances as nosodes. This has been demonstrated by Dr. Samuel Swan, of New York, who, some twelve years ago, published cures by Tuberculinum before one proving was made."

Now, Messrs. Editors, I respectfully declare that the Hahnemannian does better than "approximate the curative value" of his remedies, for he knows them through their provings. Approximate really means a close guess; knowledge, a certainty. Do we "approximate the curative value" of our grand polychrests? Should we "approximate the curative value" of any remedy when we can attain a certain knowledge by simply proving it upon the healthy, as Hahnemann did.

Again, Messrs. Editors, you tell us that "*This has been demonstrated before one proving was made.*" What has been demonstrated before one proving was made? The curative value of a homœopathic remedy? Never! Proving remedies before trying them upon the sick was the key-note of Hahnemann's great revolution in medicine. Trying them *before one proving was made* has been the old, old fallacious method of the old school these thousand years. Are we going back to it?

And worst of all, Messrs. Editors, you quote a case to prove or to show how "*this has been demonstrated before one proving was made,*" and if your reasoning was bad what shall be said of your proof! The history of a young girl is given, who had been sick since she was three years old—at the time the history begins she was twenty-one; therefore she had been sick for eighteen years. Most of these years she had been very sick, at least such is the impression one gathers from the meagre and disjointed "case" given. Such patients do not recover except under the best of care, and even then, only after years of most judicious treatment. But in this case, the patient recovers immediately after the exhibition of a dose of a remedy given upon a *supposition.* After three years of treatment, the physician strikes upon a lucky guess and cures his case in the twinkling of an eye. Truly marvelous. Did you ever make such a cure, Mr. Editor? Did you, reader?

From May to November the patient had "unconscious fits," each preceded by *a shuddering like a chill,* that seemed to go from her brain down her spine. One cannot gather from the narrative just how many of these shuddering chills the patient had, but it may fairly be presumed she had at least a dozen. "This shuddering like a chill being so like the formation of pus I [Dr. Swan] gave Tuberculinum$^{mm}$, one dose. That evening she had an attack of great violence, lasting nearly two hours, and that is *the last she had.*" This is certainly one of the most marvelous cures on record: a medicine given because the patient had shudderings like a chill which were *supposed* to be like the formation of pus. As the patient had had at least a dozen of these shudderings before, one might ask, How about the pus they heralded? A somewhat similarly rapid cure is recorded in Acts III, 1–8.

But, really, Messrs. Editors, such cases are too ridiculous to find place in any creditable journal. Just think, a remedy is *supposed* to be "good for curing pus"; it is given on the further *supposition* that pus is forming and, presto! the patient is cured. This may be true but it surely is not Homœopathy. If these nosodes are such wonderful curative agents as unproven empirical agents would they not be immensely more valuable if proven? Would our present great polychrests be such if they had not been so thoroughly proven? Using such unproven agents is a departure from the inductive method of Hahnemann. Dr. Hering said, "If our school ever gives up the strict inductive method of Hahnemann, we are lost, and deserve only to be mentioned as a caricature in the history of medicine." E. J. L.

---

["Optics sharp it takes, I ween,

To see what is not to be seen."

If we understand English we are at a loss to determine where our esteemed correspondent finds ground for the ridicule contained in the above. All that is logically embraced in his objections may be found in the last paragraph of the article to which he refers, and which he fails to quote. It is as follows:

"These two cases show what Tuberculinum is capable of doing, but we must not rest here. Until we have a thorough proving of this remedy we shall not be able to fix its place specifically. It is incumbent upon us to prove not only this but other nosodes, and then we shall be competent to say to what condition of disease they are scientifically applicable."

Our esteemed correspondent refers us to Acts III. If he will continue in that chapter he will find as much amazement expressed at that cure as is often now given utterance to at the cures made by Homœopathy, for "they were filled with wonder and amazement at that which had happened unto him."—G. H. C.]

